# Factors Defining the Functional Oligomeric State of *Escherichia coli* DegP Protease

**DOI:** 10.1371/journal.pone.0018944

**Published:** 2011-04-22

**Authors:** Jack Iwanczyk, Vivian Leong, Joaquin Ortega

**Affiliations:** 1 Department of Biochemistry and Biomedical Sciences, McMaster University, Hamilton, Ontario, Canada; 2 M.G. DeGroote Institute for Infectious Diseases Research, McMaster University, Hamilton, Ontario, Canada; Baylor College of Medicine, United States of America

## Abstract

*Escherichia coli* DegP protein is a periplasmic protein that functions both as a protease and as a chaperone. In the absence of substrate, DegP oligomerizes as a hexameric cage but in its presence DegP reorganizes into 12 and 24-mer cages with large chambers that house the substrate for degradation or refolding. Here, we studied the factors that determine the oligomeric state adopted by DegP in the presence of substrate. Using size exclusion chromatography and electron microscopy, we found that the size of the substrate molecule is the main factor conditioning the oligomeric state adopted by the enzyme. Other factors such as temperature, a major regulatory factor of the activity of this enzyme, did not influence the oligomeric state adopted by DegP. In addition, we observed that substrate concentration exerted an effect only when large substrates (full-length proteins) were used. However, small substrate molecules (peptides) always triggered the same oligomeric state regardless of their concentration. These results clarify important aspects of the regulation of the oligomeric state of DegP.

## Introduction

*Escherichia coli* DegP (also called HtrA or protease Do) is an important enzyme that contributes to the maintenance of protein homeostasis in the bacterial periplasm [1], [2], [3]. Through its combined protease and chaperone activities [4], DegP degrades or refolds misfolded and aged proteins, thereby protecting the bacterial envelope from their detrimental effects. The amount of unfolded and damaged proteins increases during cellular stress, thus DegP activity becomes essential for bacterial survival under challenging conditions [1], [5]. DegP has also been involved in preventing the cleavage of the outer-membrane proteins (OMPs) during their transit through the periplasmic space after they are secreted through the cytoplasmic membrane as unfolded polypeptide chains [6]. An interesting adaptation of DegP to the periplasmic environment lacking ATP, is that it does not require this metabolic form of energy to perform both its digestive and remodeling activities [7].

The DegP monomer contains a N-terminal chymotrypsin-like protease domain and two PDZ domains (PDZ1 and PDZ2). The three domains are linked by flexible loops. The monomers oligomerize into a hexameric cage with a central cavity where the catalytic sites are sequestered [8]. Both the top and bottom of the cage are constructed with the protease domain of three DegP monomers. Three spacing pillars connect these two trimers, each made by two intertwined loops (LA loops) extending from opposite monomers. The PDZ domains extend outward from the ends of the cage and interact at the equator of the assembly. Indeed, the stability of the hexamer is dependent on trimer-trimer interactions mediated by the LA loops and PDZ domains protruding from opposite ends of the cage [9]. In addition, each LA loop extends beyond the pillar it is forming and ends up protruding into the active site of the subunit in the opposite trimer. There, it interacts with its active site loops L1 and L2, forcing the catalytic site into a twisted inactive conformation. Therefore, the hexameric form of DegP constitutes an inactive state of the protein [8].

DegP oligomerizes into 12-mer (DegP_12_) and 24-mer (DegP_24_) globular cages in the presence of substrates. These complexes are made from either four or eight identical trimeric units, respectively [10], [11]. In the 24-mer cage the trimers are positioned at the vertices of an octahedron forming a porous shell that encloses a large inner cavity of 110 Å in diameter. Interaction between neighboring trimeric units is mediated through PDZ domains, defining several pores in the shell. These interactions are all identical and occur between the PDZ1 domain of one trimeric unit and the PDZ2 domain of the contiguous trimeric unit. In the 12-mer cage, the four trimeric units arrange in a tetrahedral shell also enclosing a central cavity. Similarly, the interaction between trimers also occurs through PDZ domains. Both structures enclose the catalytic sites within the inner cavities but differently from the hexamer they are in an active conformation, as the LA loops have been moved away and are not forcing the active site loops L1 and L2 into a twisted conformation [12].

The cascade of events through which the substrate induces the change from the inactive hexamers to the proteolytically active 12-mer and 24-mer cages has been recently described [13], [14]. On one side, the interaction of the C-terminal end of the protein substrate with the peptide-binding pocket of the PDZ1 domain causes a conformational change in this domain weakening the PDZ-PDZ domain interactions that stabilize the hexamer, thus favoring its dissociation. In this new conformation, the PDZ1 domain interacts with one of the protease loops (L3) and becomes locked into the optimal orientation to build up the 12-mer and 24-mer oligomers. Interactions between trimers in these larger oligomers are further facilitated because substrate binding also induces the displacement of a polar surface in the PDZ1 domain that leads to the exposure of a hydrophobic cleft in this domain that can now interact with a matching hydrophobic patch in the surface of the PDZ2 domains of an adjacent trimer. Simultaneously to these events the upstream sequences in the substrate molecule also bind to the proteolytic site. This interaction disturbs the interplay between the LA and the L1 and L2 loops, which in turn also deteriorates the stability of the hexamer and leaves the L1 and L2 loops free to adopt a proteolytically active conformation. Once substrate has been degraded or refolded, DegP reverts to the inactive hexameric form or it can also undergo self-degradation in a process that is allosterically stimulated by peptides resulting from substrate hydrolysis [15], [16].

In the context of this functional model, it is unclear what determines whether DegP_12_ or DegP_24_ will be assembled in the presence of a specific substrate. In this study, we found that the size of the substrate molecule is the principal determinant dictating the oligomeric state adopted by DegP. We found that substrate concentration has an effect but only with large substrate (full-length proteins), whereas small substrates (peptides) always trigger the same oligomeric form regardless of their concentration. Instead, temperature, a major regulatory factor of the activity of this enzyme [4], [17], does not influence the oligomeric form triggered by a specific substrate. These findings are discussed in the context of the recently described functional model of DegP [13], [14].

## Results

### Substrate size determines DegP oligomeric state

The large DegP_12_ and DegP_24_ cage structures of the enzyme in the presence of substrates [10], [11] confirmed early models of DegP as a self-compartmentalized protease [7], [18], and suggested that the role of the bigger cages is to accommodate larger substrates that otherwise would not fit inside the DegP hexamer. However, from these studies it is unclear why two active oligomeric forms are necessary and what determines whether DegP_12_ or DegP_24_ are assembled in the presence of a specific substrate. We hypothesized that two active oligomeric sizes are required to encapsulate substrate molecules of various sizes and that the substrate size dictates the active cage that DegP forms.

**Figure 1:**
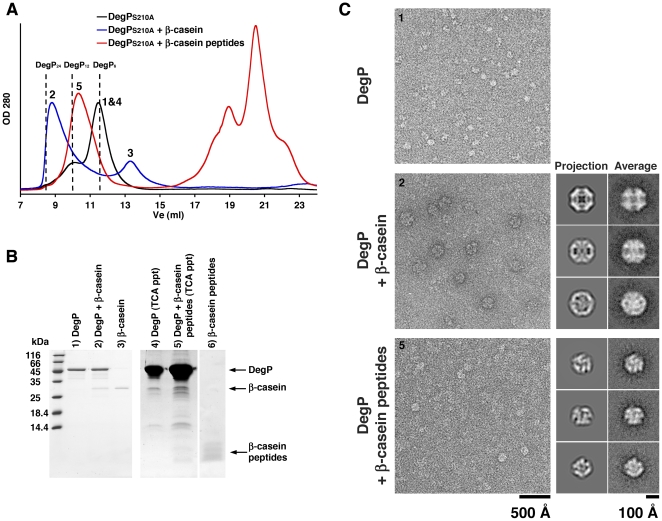
Oligomeric state of DegP_S210A_ in the presence of substrates of different sizes. **A**. Elution profiles from a Superdex-200 column of solutions containing either DegP_S210A_ alone (42 µM) (black line) or DegP_S210A_ (42 µM) mixed with β-casein (42 µM) (blue line) or β-casein peptide fragments (168 µM) (red line). The expected elution volumes (Ve) for DegP_6_ (11.5 ml), DegP_12_ (9.9 ml) and DegP_24_ (8.4 ml) are indicated by the dashed lines in the plot. Numbers above the peaks refer to the gel lanes in the SDS-PAGE gel in panel B. **B**. SDS-PAGE analysis of fractions from the elution profiles in panel A. Lane 1, 2 and 3 resolved samples from fractions indicated with the corresponding numbers in the elution profiles in panel A. Similarly, lane 4 & 5 also contained samples from fractions indicated with the corresponding number in the elution profiles in panel A. However, these fractions were previously concentrated by Trichloroacetic acid precipitation (TCA ppt) to visualize the β-casein peptides. Lane 6 shows a sample of β-casein peptides used to form complexes. **C**. The DegP_S210A_:substrate complexes purified by size exclusion chromatography in (A) were visualized by negative staining EM. The left panels shows representative negatively stained electron micrographs obtained from the central fraction of the peaks labeled as “1&4” (top), “2” (middle) and “5” in the elution profiles in (A). The right panels shows a comparison of two-dimensional projections (column labeled as “Projection”) calculated from the X-ray structure of DegP_24_ (PDB ID: 3CS0) (middle panel) or the cryo-EM structure of DegP_12_ (PDB ID: 2ZLE) (bottom panel) with class averages (column labeled as “Average”) of the corresponding views calculated from particle images extracted from the micrographs in the left panel.

Differently from studies so far that looked at oligomeric re-organization of DegP into larger 12 and 24-mer cages using substrates with varying sequences and lengths [10], [11], [13], [14], in our case, we first tested our hypothesis using substrates that only vary in length. To that end, a proteolytically inactive variant of DegP (DegP_S210A_) was assembled in the presence of either full-length β-casein, or proteolytically cleaved fragments of β-casein. Proteolytically inactive DegP_S210A_ was necessary in these assays to prevent hydrolysis of full-length β-casein. Peptide fragments were generated by proteolytically active DegP from full-length β-casein as previously shown [15]. Fragments were generated to a variety of sizes, but all of them were smaller than 10 kDa when resolved by size exclusion chromatography (Fig. 1A) or SDS-PAGE (Fig. 1B; lane 6).

The oligomeric state of the DegP_S210A_:substrate complexes formed in the presence of either full-length β-casein or its fragments was monitored by size exclusion chromatography. Consistent with previous studies, DegP_S210A_ alone eluted at a position expected for a hexamer [9] and in the presence of full-length β-casein we observed a peak eluting at the expected elution volume for DegP_24_ cages (Fig. 1A) [10], [11]. However, when the small β-casein fragments were used as substrate, DegP_S210A_ eluted to a size corresponding to DegP_12_ cages (Fig. 1A). Samples withdrawn from each peak and resolved by SDS-PAGE confirmed the presence of DegP_S210A_ and substrate in these peaks (Fig. 1B).

**Figure 2:**
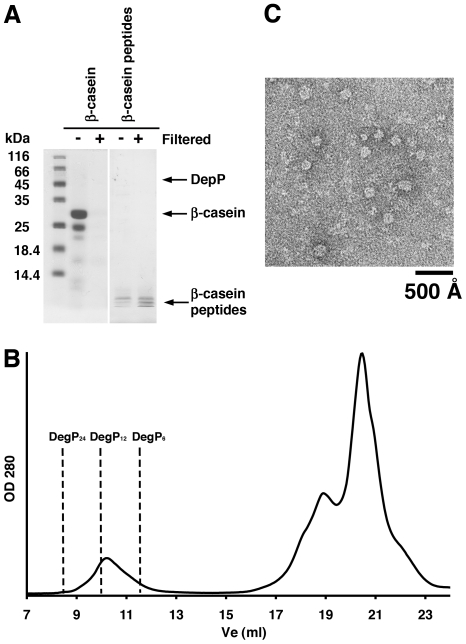
Filtered β-casein peptide fragments induce DegP_12_ cages. **A**. A 15% SDS-PAGE resolving a preparation of full-length β-casein (lines labeled as “β-casein”) and β-casein peptide fragments (lines labeled as “β-casein peptides”) before (−) and after (+) they were filtered through a 30 kDa-cutoff filter to remove full-length β-casein. Lines containing full-length β-casein were stained with Coomassie brilliant blue and lines containing the β-casein peptide fragments were silver stained to visualize that no full-length β-casein or proteolytically active DegP remained after filtration. **B**. Elution profiles from a Superdex-200 column of solutions containing DegP_S210A_ (42 µM) mixed with a preparation of β-casein peptide fragments (168 µM) that has been filtered through a 30 kDa-cutoff filter to remove traces of full-length β-casein. The expected elution volumes (Ve) for DegP_6_ (11.5 mL), DegP_12_ (9.9 mL) and DegP_24_ (8.4 mL) are indicated by the dashed lines in the plot. **C**. Negatively stained electron micrograph obtained from the fraction at 10 mL from the size exclusion chromatography profile shown in panel B.

To rule out the possibility that traces of full-length β-casein remaining in the preparation of peptide fragments were inducing the observed DegP_12_ cages, the sample was filtered through a 30 kDa-cutoff filter. Full-length β-casein has an apparent molecular size of ∼130 kDa as seen by size exclusion chromatography (Fig. 1A) and it was efficiently removed by this procedure based on the following experiments. First, when the same concentration of undigested β-casein was filtered, approximately 98% of the full-length protein was retained (Fig. 2A) and the remaining amount was not sufficient to trigger any oligomerization changes to DegP (data not shown). Second, the preparation of β-casein peptides used to trigger the assembly of large oligomers did not show any traces of full-length β-casein by silver staining SDS-PAGE after passing it through the filter (Fig. 2A), but it still triggered the formation of DegP_12_ cages (Fig. 2B). The filtering procedure also removed any residual amounts of proteolytically active DegP in the peptide preparation (Fig. 2A). Therefore, these results confirm that in the preparation of hydrolyzed β-casein, the peptides were the component responsible for triggering the assembly of DegP_12_ cages rather than any residual amount of full-length β-casein.

To corroborate that properly assembled DegP_6_, DegP_24_ and DegP_12_ cages were present in the corresponding peaks of the elution profiles, fractions from these peaks were visualized by electron microscopy under negative-staining conditions (Fig. 1C and 2C). Images from the peak at the expected elution volume for a hexamer showed globular structures consistent in size with the dimensions of hexameric cages (Fig. 1C, top panel). Similarly, EM images were obtained from the peaks corresponding to 24 and 12-mer DegP oligomers (Fig. 1C, left middle and bottom panels and Fig. 2C). In both cases, EM images showed large structures closely resembling the previously described DegP_24_ and DegP_12_ cages, respectively [10], [11]. Classification of the particle images obtained in each condition using 2D projections calculated from the available DegP_24_ (PDB ID: 3CS0) and DegP_12_ (PDB ID: 2ZLE) cage structures, allowed us to obtain two-dimensional averages representing typical projections of these structures, confirming the identity of these particle images (Fig. 1C, right middle and bottom panels).

**Figure 3:**
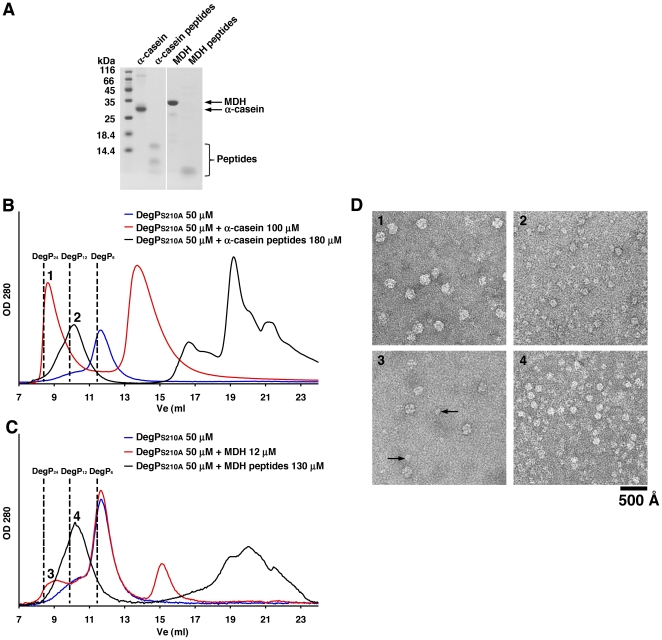
Oligomeric state of DegP_S210A_ in the presence of α-casein, malate dehydrogenase and their corresponding peptide fragments. **A**. Coomassie brilliant blue stained 15% SDS-PAGE resolving the full-length α-casein and malate dehydrogenase (MDH) used to induce the formation of large oligomeric states in DegP_S210A_. Peptides generated from both proteins by incubation with DegP are also shown in the gel to provide an estimation of their molecular weight. **B**. Elution profiles from a Superdex-200 column of solutions containing either DegP_S210A_ alone or DegP_S210A_ mixed with α-casein or α-casein peptide fragments. The concentration of each component of the reaction is indicated. The expected elution volumes (Ve) for DegP_6_ (11.5 ml), DegP_12_ (9.9 ml) and DegP_24_ (8.4 ml) are indicated by the dashed lines in the plot. Numbers above the peaks refer to the electron micrographs in panel D. **C**. Elution profiles from a Superdex-200 column of solutions containing either DegP_S210A_ alone or DegP_210A_ mixed with MDH or MDH peptide fragments. The expected elution volumes (Ve) for DegP_6_ (11.5 ml), DegP_12_ (9.9 ml) and DegP_24_ (8.4 ml) are indicated by the dashed lines in the plot. Numbers above the peaks refer to the electron micrographs in panel D. **D**. Visualization of complexes of DegP_S210A_ with α-casein and MDH substrates purified by size exclusion chromatography. The images are representative fields of negatively stained electron micrographs obtained from fractions labeled from 1 to 4 in the elution profiles shown in panels B and C. The arrowheads in panel 3 indicate some DegP_12_ cages present in this fraction.

To generalize our conclusion, a similar analysis was repeated with four additional substrates: two full-length proteins, α-casein and malate dehydrogenase (MDH) and their corresponding peptide fragments generated by proteolytically active DegP. As determined by SDS-PAGE (Fig. 3A) and size exclusion chromatography (Fig. 3B and 3C) the peptide fragments obtained from α-casein and MDH were smaller than 16 and 10 kDa, respectively. Similarly to the results obtained with β-casein, both full-length proteins induced the formation of mainly DegP_24_ cages and the two preparations of peptide fragments prepared from these proteins triggered the assembly of DegP_12_ oligomers only (Fig. 3B and 3C). Negative stained electron micrographs collected from the peaks of the size exclusion chromatography profile corresponding to the 24 and 12-mer DegP oligomers corroborated the identity of these complexes. We noticed that in the elution profile of the mixture containing DegP_S210A_ and full-length MDH the peak corresponding to the 24-mer oligomers was smaller and slightly shifted towards a lower molecular weight (Fig. 3C). The electron micrographs confirmed that these fractions mainly contained DegP_24_ cages however; a few DegP_12_ cages were also present (Fig. 3D; arrows). The size exclusion chromatography profile also presented a large peak at the elution volume corresponding to hexamers and an additional peak at the elution volume corresponding to free MDH (Fig. 3C) indicating that a big proportion of the enzyme still remained in the inactive state and it did not interact with the substrate. We attributed the lower capacity of MDH to trigger the assembly 24-mer cages and to produce small amounts of 12-mer cages to the fact that compared to β-casein, MDH is poorly recognized by DegP_S210A_ as a substrate [9]. This result is consistent with our finding described below that large substrates trigger either DegP_24_ or DegP_12_ cages depending on their concentration.

**Figure 4:**
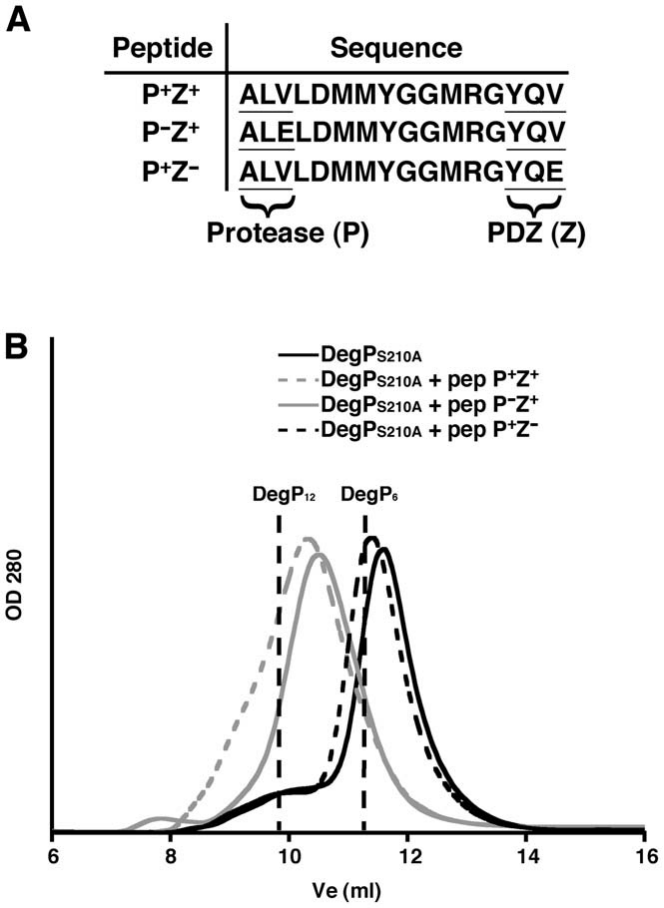
Oligomeric state of DegP_S210A_ in the presence of PZ model peptides. **A**. Names and sequences of the three PZ model peptides used to form complexes with DegP_S210A_ in the size exclusion chromatography experiment shown in (B). Each peptide contains a protease motif at the N-terminus (labeled as “P”) and a PDZ motif at the C-terminus (labeled as “Z”). The +/− sign next to the “P” and “Z” labels indicated whether the motif is recognized (+) or not recognized (−) by the corresponding domain. **B**. Elution profiles from a Superdex-200 column of solutions containing DegP_S210A_ alone (94 µM) or a mixture of DegP_S210A_ (94 µM) with one of the model peptides at a concentration of 376 µM. The expected elution volumes (Ve) for DegP_6_ (11.5 ml) and DegP_12_ (9.9 ml) are indicated by the dashed lines in the plot.

To show whether peptides that have not being generated by proteolytically active DegP also have the ability to induce DegP_12_ cages, we synthesized three peptides (Fig. 4A). The first peptide contained the sequence motif “ALV” at the N-terminus and the sequence motif “YQV” at the C-terminus. The “ALV” N-terminal motif is recognized by DegP protease domain and the “YQV” C-terminal motif binds to the DegP PDZ1 domain [12]. Therefore, these sequences were given the name of P (for protease) and Z (for PDZ) motifs, respectively. In this peptide the 10-residue linker “LDMMYGGMRG” corresponding to a non-cleavable segment of citrate synthase connects the P and Z motifs. This entire peptide was called P^+^Z^+^ (Fig. 4A), being the “+” sign to indicate that DegP recognizes both motifs through its protease and PDZ1 domains, respectively. The second peptide, named P^−^Z^+^, was identical to the first one but the P motif was altered from “ALV” to “ALE” (Fig. 4A) preventing its cleavage by DegP but retaining its PDZ1 binding ability [12] . Finally the last peptide was named P^+^Z^−^, since it maintained the motif “ALV” in the P motif but contained the modified sequence “YQE”, instead of “YQV”, in the Z motif (Fig. 4A) and thus missing its binding ability to the PDZ1 domain [12].

When DegP_S210A_ was incubated in the presence of the P^+^Z^+^ peptide containing both a protease binding motif and a PDZ1 binding motif, the enzyme eluted as a 12-mer oligomeric cage upon injection into the filtration column (Fig. 4B), consistent with rearrangement in the presence of small substrates. A similar experiment with the P^−^Z^+^ peptide that maintained binding to the PDZ1 domain produced a peak in the elution profile at the expected elution volume of DegP_12_ cages (Fig. 4B). Conversely, the P^+^Z^−^ peptide that is not bound by the PDZ1 domain [12] was not sufficient to trigger formation of DegP_12_ (Fig. 4B) corroborating [13] that the interaction of the C-terminal end of the substrate with the PDZ1 domain is responsible for triggering the reorganization of hexameric DegP into DegP_12_ cages. The interaction of upstream sequences with the protease domain was not able to initiate this process. These experiments show that both DegP generated and synthetic peptides have equal ability to induce formation of DegP_12_ cages.

All together these results confirm our hypothesis that DegP is able to sense the size of the substrate molecules and adapt its oligomeric state to encapsulate substrate of various sizes.

### Effect of substrate concentration in DegP oligomeric state

**Figure 5:**
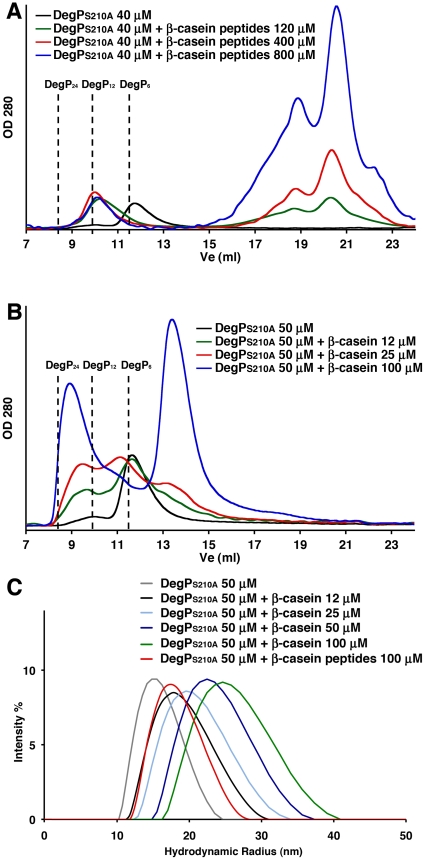
Oligomeric state of DegP_S210A_ at various substrate concentrations. Elution profiles of DegP_S210A_ in the presence of various concentrations of β-casein peptides (A) or full-length β-casein (B). The expected elution volumes (Ve) for DegP_6_ (11.5 ml), DegP_12_ (9.9 ml) and DegP_24_ (8.4 ml) are indicated by the dashed lines in the plot. (C) Dynamic light scattering analysis of DegP_S210A_ in the presence of variable concentrations of full-length β-casein or β-casein peptides. The graph shows the distribution of hydrodynamic radius of particles for various mixtures of DegP_S210A_ and full-length β-casein or DegP_S210A_ and β-casein peptides.

To determine whether the substrate concentration has any effect in the oligomeric state adopted by DegP, we repeated the size exclusion chromatography experiment by mixing DegP_S210A_ with several concentrations of β-casein peptides and full-length β-casein. Interestingly, β-casein peptides consistently induced DegP_12_ cages even when mixed in large molar excess (20×; with respect to DegP_S210A_ monomers) (Fig. 5A). However, full-length β-casein triggered formation of DegP_24_ cages when the substrate was in molar excess but at substoichiometric amounts the peak in the elution profile shifted towards the elution volume expected for DegP_12_ cages (Fig. 5B) suggesting that a mixture of 12 and 24-mer oligomeres was formed.

We confirmed the presence of both oligomeric states in the mixture containing substoichiometric amounts of substrate by dynamic light scattering (DLS) (Fig. 5C). In this experiment, we first analyzed homogeneous control samples containing DegP_6_ (DegP_S210A_ alone), DegP_12_ (DegP_S210A_ with β-casein peptides) or DegP_24_ cages (DegP_S210A_ with full-length β-casein in molar excess). The sample containing mainly hexamers (DegP_S210A_ alone) showed a peak suggesting that the hydrodynamic radii of these particles ranges from 10–24 nm with most of the protein at 15 nm. DegP_S210A_ with a large molar excess of β-casein peptides, containing primarily 12-mer oligomers produced a peak centered at 17 nm and indicated that the hydrodynamic radii of these cages vary from 12–27 nm. Finally, the last control sample, DegP_S210A_ with full-length β-casein in molar excess, containing only 24-mer cages, produced a broader peak signifying that the hydrodynamic radii of these particles extends from 16–40 nm with most of the particles at 24 nm. These obtained hydrodynamic radii measurements agreed well with previously reported dimensions of the oligomers [8], [10], [11]. Finally, consistent with the results obtained by size exclusion chromatography experiment (Fig. 5B), when two mixtures containing DegP_S210A_ and either equimolar or substoichiometric amounts of full-length β-casein were measured by DLS, the obtained peaks showed a distribution of hydrodynamic radii between the one obtained for the sample containing exclusively DegP_24_ cages and the one containing only DegP_12_ cages (Fig. 5D). These results imply that small substrates (peptides) always trigger the same oligomeric form regardless of their concentration. However, in the presence of large substrates (full-length proteins) the substrate concentration has an effect in the oligomeric state adopted by DegP.

### Temperature does not affect the substrate-determined DegP oligomeric state

Temperature was early on recognized as an important factor conditioning which of the two DegP activities (protease or chaperone) is exerted upon the substrate molecule [4]. In addition, the rate of conversion from DegP_6_ to DegP_12_ and DegP_24_ cages is also temperature dependent and increases at elevated temperatures [14]. Therefore, we decided to test whether temperature influences the final oligomeric state triggered by a specific substrate.

**Figure 6:**
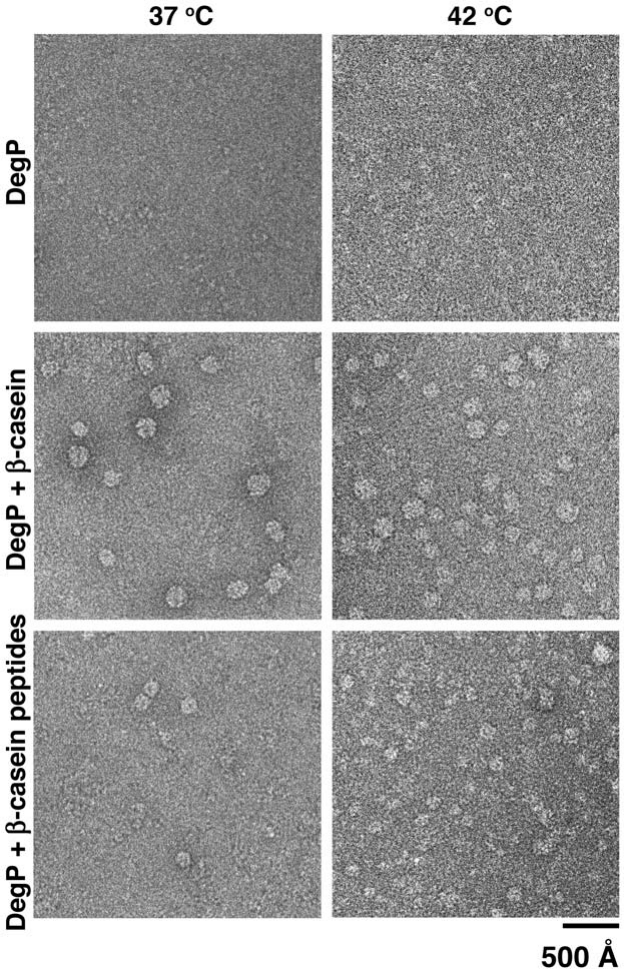
Effect of temperature on the oligomeric state of DegP_S210A_:substrate complexes. Representative electron micrographs of reaction mixtures containing DegP_S210A_ alone (top panels), DegP_S210A_+β-casein (middle panels) or DegP_S210A_+β-casein peptides (bottom panels) at either 37°C (left panels) or 42°C (right panels). Complexes were assembled, applied to continuous carbon grids and negatively stained at the indicated temperatures.

Using negative staining, EM images of DegP_S210A_ in the absence of substrate molecules incubated at 37°C showed small globular particles consistent in size with hexameric cages. The same specimen imaged at 42°C also showed numerous small particles similar to the ones observed at 37°C (Fig. 6, top panels). It has been described that DegP_6_, DegP_12_ and DegP_24_ cages remain stable at temperatures as high as 37°C. However, the hexameric form seems to break down into trimers at 42°C [10], [11]. Our EM images were not able to resolve between particle sizes corresponding to trimer and hexamer, thus it was not possible to confirm by this method whether DegP_S210A_ was a trimer at 42°C, as previously published [10], [11]. It was clear however, that these particles (Fig. 6, top panels) were smaller than either DegP_12_ or DegP_24_ cages (Fig. 6, middle and bottom panels). In the presence of either full-length β-casein (Fig. 6, middle panels) or β-casein peptides (Fig. 6, bottom panels), DegP_S210A_ assembled as DegP_24_ or DegP_12_ cages respectively, regardless of the temperature.

**Figure 7:**
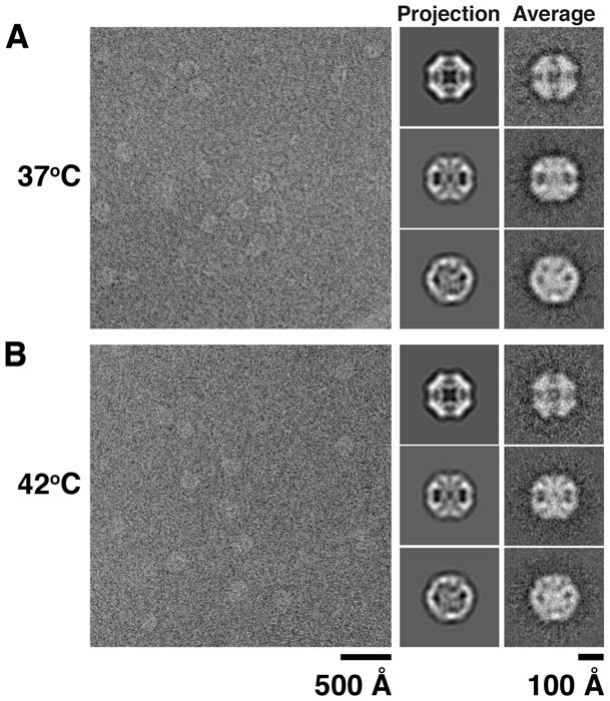
Cryo-electron microscopy of a reaction mixture containing DegP_S210A_ and β-casein at high temperatures. Representative micrographs of vitrified samples of a mixture of DegP_S210A_ and β-casein at 37°C (A) and 42°C (B). Contrast of both images was inverted for easy comparison with negatively stained micrographs in previous figures. The right panels in (A) and (B) show a comparison of two-dimensional projections (column labeled as “Projection”) calculated from the X-ray structure of DegP_24_ (PDB ID: 3CS0) with class averages (column labeled as “Average”) of the corresponding views calculated from particle images extracted from the micrographs.

Consistent with our size exclusion chromatography results, most particles in the DegP_S210A_+β-casein peptides sample classified as 12-mer oligomers (∼85%) using a multireference classification approach (see Experimental Procedures). However, one caveat on these experiments was that many particles in the DegP_S210A_+β-casein sample (∼60%) were misclassified and not recognized as 24-mer oligomers, since they were collapsed or distorted as a result of the dehydration inferred during the negative staining procedure [19]. To resolve this issue, the mixture of DegP_S210A_ and full-length β-casein was visualized under cryo-electron microscopy conditions in the absence of stain at either 37°C (Fig. 7A) or 42°C (Fig. 7B). At either temperature most particles were classified as DegP_24_ cages (∼80%). Two-dimensional class averages from particle images obtained at both 37°C (Fig. 7A, right panel, column labeled as “Average”) and 42°C (Fig. 7B, right panel, column labeled as “Average”) resembled corresponding projection views calculated from the X-ray structure of DegP_24_ cage (Fig. 7A and 7B, right panels, column labeled as “Projection”) [10], [11] confirming the identity of these particles. These data indicate that temperature does not have an effect on the oligomeric state triggered by a substrate molecule in DegP.

## Discussion

Recent work from several groups has shown that the presence of substrate triggers the reorganization of inactive DegP hexamers into 12 and 24-mer oligomeric cages that are proteolytically active [10], [11]. The study presented here asks what determines whether DegP_12_ or DegP_24_ cages are assembled. We found that the size of the substrate molecule is the main determinant of the functional oligomeric state adopted by the enzyme.

Interestingly, the concentration of substrate had an effect in the oligomeric state triggered by the substrate depending on its size. Small substrates (peptides) always triggered DegP_12_ cages regardless of the concentration used (Fig. 5A) but large substrates (full-length proteins) produced DegP_24_ cages when the substrate was added in molar excess or a mixture of DegP_24_ and DegP_12_ cages in the presence substoichiometric amounts of substrate (Fig. 5B and 5C). Our analysis by SDS-PAGE of the DegP-substrate complexes purified using size exclusion chromatography (Fig. 1B) provided an explanation for these results. We observed that regardless of the size or concentration of substrate in the reaction, the purified DegP-substrate complexes always contained substoichiometric amounts of substrate. This result indicates that large DegP cages only enclose a few substrate molecules at a time, even in the presence of a large molar excess of substrate. Consequently, it is not possible to accumulate enough of a particularly small substrate, such as the β-casein peptides, inside DegP_12_ cages to completely fill up the inner cavity and thus trigger reorganization of the enzyme into larger DegP_24_ cages. Conversely, DegP forms 24-mer cages to enclose several molecules of full-length protein in the presence of a molar excess of substrate. However, lowering the substrate concentration decreases the number of molecules enclosed in the cages and below a certain number, DegP_12_ cages are sufficient to compartmentalize the substrate.

Rather than simply the substrate molecular mass, our results presented here indicate that at a specific substrate concentration, it is most likely the combination of the number of substrate molecules bound to the DegP trimers forming the cage and the apparent size of the substrate what determines the functional oligomeric state reached by DegP. Considering these factors, it is not surprising that Krojer *et al.* [11] found that the inner chamber of DegP_12_ was large enough to encapsulate a folded 38-kDa outer membrane protein. However, we observed that β-casein with a molecular mass of 29 kDa, triggered the formation of DegP_24_ cages when present in molar excess (Fig. 1A and 5). This results is due to the large apparent size of β-casein, which is ∼120 kDa as measured by size exclusion chromatography (Fig. 1A). Consequently, if at a specific substrate concentration several β-casein molecules bind to the DegP trimers forming the cage, and each β-casein molecule takes on a size several times larger than its molecular weight, then DegP_12_ will not be large enough and DegP_24_ cages will be required to enclose the substrate.

Temperature has been recognized as a critical factor regulating several aspects of the protease/chaperone activities of DegP [4]. Krojer et al. [13] identified the LA loop in DegP as a temperature-sensing motif. Raising the temperature causes an increase in the flexibility of the LA loop weakening the trimer-trimer interactions in DegP_6_ and thus, facilitating the formation of DegP_12_ and DegP_24_ cages. In addition, it has been described that the rate of conversion from DegP_6_ to DegP_12_ and DegP_24_ cages is temperature dependent and increases at elevated temperatures [14]. These results indicate that temperature plays an important role in initiating the substrate-induced oligomeric rearrangement of DegP and also in the rate of conversion between oligomeric states. However, our experiments show that this factor does not influence the final oligomeric state adopted by DegP in the presence of a specific substrate.

Surprisingly, a recent study to characterize the mechanisms whereby peptidic activators trigger a change in oligomeric state [14] suggested that DegP assembles as a 24-mer oligomer in the presence of 7–10 residue long synthetic peptides. This finding is clearly in contradiction with our results shown herein (Fig. 4). The purpose of the study by Merdanovic et al. [14] was to reveal the local rearrangements accompanying the binding of the substrate to the PDZ1 domain, rather than to characterize whether these small substrates trigger either DegP_12_ or DegP_24_ cages. To this end, crosslinking and electrophoresis was used to differentiate the oligomeric state adopted by the enzyme. This method properly differenciates between DegP_6_ and higher order oligomers but it has a limited capability to differentiate between DegP_12_ and DegP_24_ cages. Indeed, in the same study Merdanovic et al. [14] also analyzed a mixture of DegP and β-casein peptides by analytical centrifugation and found that DegP_12_ cages was the most prominent oligomeric form in the mixture, which is in agreement with our results presented here. Here, we used size exclusion chromatography in our experiments that has resolving power superior to electrophoresis and is able to clearly differentiate between the three main oligomeric states adopted by the enzyme. Therefore, we believe that the inability to differentiate between 12 and 24-mer oligomers by electrophoresis is at the root of the disagreement between these two studies.

In summary, our findings provide additional details of the factors regulating the process of oligomeric reorganization of DegP from inactive hexamers to functionally active larger cages in the presence of substrate molecules. In particular, this work suggests that the role of the larger oligomeric states of DegP (DegP_12_ and DegP_24_) is to accommodate substrates of various sizes.

## Materials and Methods

### Plasmids and mutagenesis

The pET21b-HtrA and pET21b-HtrA_S210A_ plasmids encoding DegP and the proteolytically inactive DegP_S210A_, respectively were obtained as described previously [9].

### Protein expression and purification

DegP and the proteolytically inactive DegP_S210A_ proteins were expressed and purified as C-terminal His-tagged constructs according to previously published methods [9].

### Peptide substrates

Peptide substrates from β-casein, α-casein and Malate Dehydrogenase (MDH) were generated and purified following a previously described method [15]. Briefly, substrate hydrolysis reactions had a volume of 250 µL and they were prepared in 50 mM HEPES, pH 7.3. These reactions contained 5 µM DegP and 86 µM of β-casein or α-casein (Sigma). In the case of Malate Dehydrogenase (MDH) the concentrations of DegP and MDH were 1.3 µM and 13 µM, respectively. Reactions were incubated at 37°C for 1 hour (β-casein) or 37°C for 2 hours (α-casein) or for 4 hours at 42°C (MDH). Subsequently, they were heated at 95°C for 10 min and immediately chilled on ice for 10 min to terminate the reaction and precipitate DegP and remaining undigested full length substrate. Samples were spun down at 12,000 rpm in a bench-top centrifuge at 4°C to remove the precipitate. The supernatants containing the peptides were concentrated by Savant DNA120 SpeedVac and were used for further reactions. Peptide concentrations were estimated considering the total amount of full-length substrate that was used in the hydrolysis reaction and the average molecular weight for the resulting peptides obtained from Coomassie brilliant blue stained SDS-PAGE gels.

The three PZ model peptides were designed according to a previous study [12] and synthesized by GenScript USA Inc. (Piscataway, NJ, USA). The sequences and names of the three peptides are as follows: P^+^Z^+^ ALVLDMMYGGMRGYQV; P^−^Z^+^ ALELDMMYGGMRGYQV; P^+^Z^−^ ALVLDMMYGGMRGYQE.

To perform our experiments the P^−^Z^+^ and P^+^Z^−^ lyophilized peptides were dissolved to a final concentration of 5 mg/ml in 15% (v/v) Dimethyl sulfoxide (DMSO), 0.12 M NH_4_OH, 50 mM HEPES, pH 7.3. The more hydrophobic peptide PepP^+^Z^+^, was dissolved to a final concentration of 20 mg/ml in 6 M Guanidinium chloride (GdCl) and 50 mM HEPES, pH 7.3. During reactions with DegP_S210A_, the final concentrations of Guanidinium chloride, DMSO and NH_4_OH were less than 0.06 M, 0.75% and 6 µM, respectively and these amounts did not significantly alter the integrity of DegP oligomers as seen by size exclusion chromatography (Fig. 4B).

### Size exclusion chromatography

Size exclusion chromatography experiments to analyze DegP:substrate complexes were performed on a Superdex 200 10/300 GL column (GE Healthcare Life Sciences). The column was equilibrated in 50 mM HEPES, pH 7.3, 150 mM NaCl at 4°C. Purified DegP_S210A_ was incubated in 50 mM HEPES, pH 7.3 at 37°C for 10 min, either alone or with the specified substrates. The concentration of DegP_S210A_ and substrate for each specific experiment is indicated in the corresponding figure or figure legend. In all cases, the concentrations of DegP were calculated using molecular weight of the monomer. Before the mixture was loaded into the filtration column, they were spun down at 12,000 rpm in a bench-top centrifuge at 4°C. Finally, 250 µL of supernatant was injected to the column. A gel filtration calibration kit (HMW, GE Healthcare Life Sciences) was used for column calibration.

Where indicated, fractions from size exclusion chromatography experiments were precipitated by trichloroacetic acid (TCA). In these cases, 125 µL TCA was added to 500 µL samples from the fractions. Samples were incubated at 4°C for 30 minutes, then spun down at 12,000 rmp in a bench-top centrifuge at 4°C. Pellets were dried at 25°C and resuspended in 20 µL loading buffer before resolving them by SDS-PAGE.

### Dynamic light scattering

Dynamic Light Scattering (DLS) measurements were performed in a Zetasizer Nano S instrument with Zetasizer 6.01 data analysis software (Malvern. USA). Measurements were taken with a 30% laser power at a wavelength of 633 nm at a temperature of 37°C and the sample was contained in a 12 µL micro-cuvette. Protein samples containing 50 µM of DegP_S210A_ (monomer) were incubated in 50 mM HEPES, pH 7.3 at 37°C for 10 min either alone or with full-length β-casein or β-casein peptides at concentrations indicated in the corresponding figure. Each mixture was spun down at 12,000 rpm in a bench-top centrifuge before the measurement was taken. Scattered light intensity was recorded every 10 s. The number of measurements was adjusted automatically and ranged between 12 to 20. Translational diffusion coefficients were estimated from these measurements and used to calculate the hydrodynamic radii using the software provided with the instrument.

### Electron microscopy

Fractions from the filtration column were directly visualized by negative stain transmission electron microscopy. To this end, freshly glow-discharged continuous carbon grids were floated on a 5 µL drop of a fraction from the filtration column for 2 min. Excess sample was blotted and the grids were stained with 2% uranyl actetate (Canemco and Marivac) for 1 min.

In those cases were negative staining visualization of DegP cages at a specific temperature (37 or 42°C) was required a mixture containing 84 µM β-casein or β-casein peptides and 21 µM DegP was incubated at either 37 or 42°C for 10 minutes. Then a 3.6 µL drop was added to the grid maintained at either 37 or 42°C inside a Vitrobot (FEI) blotting chamber. Blotting of the grid to remove the excess of sample was done also within the Vitrobot (FEI) chamber and the filter paper used in the blotting and the 2% uranyl actetate solution were also preheated to either 37 or 42°C before applying it to the grid.

Similar samples were also visualized by cryo-EM at 37°C or 42°C. In this case freshly glow-discharged holey carbon grids previously washed with acetone vapor for 15 min were mounted in the Vitrobot (FEI) chamber equilibrated at either at 37°C or 42°C and 100% relative humidity. The mixture containing 21 µM DegP and 84 µM β-casein was incubated at either 37 or 42°C for 10 minutes and then a 3.6 µL drop was added to the grid. Subsequently, the grid was blotted and plunged into liquid ethane to vitrify the specimen. Finally, the grid was transferred to the electron microscope using a 914 Gatan Nitrogen cooled cryo-holder (Gatan Inc., Pleasanton, CA. USA).

Specimens were imaged at a nominal magnification of 50,000× in a JEOL 2010F electron microscope operated at 200 kV under low dose conditions (∼10 e^−^/Å^2^). EM images were recorded on Kodak S0-163 film and digitized with a step size of 12.7 µm in a Nikon Supercool Scan 9000 producing images with a sampling value of 2.54 Å/pixel.

### Single-particle image processing

Particles were selected interactively from the micrographs using Boxer program (EMAN) [20] and classified according to their oligomeric state and orientation by multi-reference projection matching based methods using SPIDER [21]. The three reference structures used in the multi-reference classification procedure were the X-ray structures of the DegP hexamer (PDB ID:1KY9) and DegP_24_ (PDB ID: 3CS0) and the cryo-EM structure of DegP_12_ (PDB ID: 2ZLE). Projections representing unique orientations of each of the three models were generated from the three references. Particles were compared and assigned to these projections using cross correlation. Finally, averages were generated for selected classes using reference free alignment procedures as implemented in the XMIPP software package [22].

## References

[1] Lipinska B, Fayet O, Baird L, Georgopoulos C (1989). Identification, characterization, and mapping of the Escherichia coli htrA gene, whose product is essential for bacterial growth only at elevated temperatures.. J Bacteriol.

[2] Lipinska B, Sharma S, Georgopoulos C (1988). Sequence analysis and regulation of the htrA gene of Escherichia coli: a sigma 32-independent mechanism of heat-inducible transcription.. Nucleic Acids Res.

[3] Strauch KL, Johnson K, Beckwith J (1989). Characterization of degP, a gene required for proteolysis in the cell envelope and essential for growth of Escherichia coli at high temperature.. J Bacteriol.

[4] Spiess C, Beil A, Ehrmann M (1999). A temperature-dependent switch from chaperone to protease in a widely conserved heat shock protein.. Cell.

[5] Skorko-Glonek J, Wawrzynow A, Krzewski K, Kurpierz K, Lipinska B (1995). Site-directed mutagenesis of the HtrA (DegP) serine protease, whose proteolytic activity is indispensable for Escherichia coli survival at elevated temperatures.. Gene.

[6] Misra R, CastilloKeller M, Deng M (2000). Overexpression of protease-deficient DegP(S210A) rescues the lethal phenotype of Escherichia coli OmpF assembly mutants in a degP background.. J Bacteriol.

[7] Clausen T, Southan C, Ehrmann M (2002). The HtrA family of proteases: implications for protein composition and cell fate.. Mol Cell.

[8] Krojer T, Garrido-Franco M, Huber R, Ehrmann M, Clausen T (2002). Crystal structure of DegP (HtrA) reveals a new protease-chaperone machine.. Nature.

[9] Jomaa A, Damjanovic D, Leong V, Ghirlando R, Iwanczyk J (2007). The Inner Cavity of Escherichia coli DegP Protein is not Essential for Molecular Chaperone and Proteolytic Activity.. J Bacteriol.

[10] Jiang J, Zhang X, Chen Y, Wu Y, Zhou ZH (2008). Activation of DegP chaperone-protease via formation of large cage-like oligomers upon binding to substrate proteins.. Proc Natl Acad Sci U S A.

[11] Krojer T, Sawa J, Schafer E, Saibil HR, Ehrmann M (2008). Structural basis for the regulated protease and chaperone function of DegP.. Nature.

[12] Krojer T, Pangerl K, Kurt J, Sawa J, Stingl C (2008). Interplay of PDZ and protease domain of DegP ensures efficient elimination of misfolded proteins.. Proc Natl Acad Sci U S A.

[13] Krojer T, Sawa J, Huber R, Clausen T (2010). HtrA proteases have a conserved activation mechanism that can be triggered by distinct molecular cues.. Nat Struct Mol Biol.

[14] Merdanovic M, Mamant N, Meltzer M, Poepsel S, Auckenthaler A (2010). Determinants of structural and functional plasticity of a widely conserved protease chaperone complex.. Nat Struct Mol Biol.

[15] Jomaa A, Iwanczyk J, Tran J, Ortega J (2009). Characterization of the autocleavage process of the Escherichia coli HtrA protein: implications for its physiological role.. J Bacteriol.

[16] Skorko-Glonek J, Zurawa D, Tanfani F, Scire A, Wawrzynow A (2003). The N-terminal region of HtrA heat shock protease from Escherichia coli is essential for stabilization of HtrA primary structure and maintaining of its oligomeric structure.. Biochim Biophys Acta.

[17] Sobiecka-Szkatula A, Polit A, Scire A, Gieldon A, Tanfani F (2009). Temperature-induced conformational changes within the regulatory loops L1-L2-LA of the HtrA heat-shock protease from Escherichia coli.. Biochim Biophys Acta.

[18] Kim KI, Park SC, Kang SH, Cheong GW, Chung CH (1999). Selective degradation of unfolded proteins by the self-compartmentalizing HtrA protease, a periplasmic heat shock protein in Escherichia coli.. J Mol Biol.

[19] Ruprecht J, Nield J (2001). Determining the structure of biological macromolecules by transmission electron microscopy, single particle analysis and 3D reconstruction.. Prog Biophys Mol Biol.

[20] Ludtke SJ, Baldwin PR, Chiu W (1999). EMAN: semiautomated software for high-resolution single-particle reconstructions.. J Struct Biol.

[21] Shaikh TR, Gao H, Baxter WT, Asturias FJ, Boisset N (2008). SPIDER image processing for single-particle reconstruction of biological macromolecules from electron micrographs.. Nat Protoc.

[22] Marabini R, Masegosa IM, San Martin MC, Marco S, Fernandez JJ (1996). Xmipp: An Image Processing Package for Electron Microscopy.. J Struct Biol.

